# Kar3Vik1 Uses a Minus-End Directed Powerstroke for Movement along Microtubules

**DOI:** 10.1371/journal.pone.0053792

**Published:** 2013-01-14

**Authors:** Julia Cope, Katherine C. Rank, Susan P. Gilbert, Ivan Rayment, Andreas Hoenger

**Affiliations:** 1 The Boulder Laboratory for 3-D Microscopy of Cells, Department of Molecular, Cellular, and Developmental Biology, University of Colorado, Boulder, Colorado, United States of America; 2 Department of Biochemistry, University of Wisconsin, Madison, Wisconsin, United States of America; 3 Department of Biology and the Center for Biotechnology and Interdisciplinary Studies, Rensselaer Polytechnic Institute, Troy, New York, United States of America; Helmholtz Centre for Infection Research, Germany

## Abstract

We have used cryo-electron microscopy (cryo-EM) and helical averaging to examine the 3-D structure of the heterodimeric kinesin-14 Kar3Vik1 complexed to microtubules at a resolution of 2.5 nm. 3-D maps were obtained at key points in Kar3Vik1’s nucleotide hydrolysis cycle to gain insight into the mechanism that this motor uses for retrograde motility. In all states where Kar3Vik1 maintained a strong interaction with the microtubule, we found, as observed by cryo-EM, that the motor bound with one head domain while the second head extended outwards. 3-D reconstructions of Kar3Vik1-microtubule complexes revealed that in the nucleotide-free state, the motor’s coiled-coil stalk points toward the plus-end of the microtubule. In the ATP-state, the outer head is shown to undergo a large rotation that reorients the stalk ∼75° to point toward the microtubule minus-end. To determine which of the two heads binds to tubulin in each nucleotide state, we employed specific Nanogold®-labeling of Vik1. The resulting maps confirmed that in the nucleotide-free, ATP and ADP+Pi states, Kar3 maintains contact with the microtubule surface, while Vik1 extends away from the microtubule and tracks with the coiled-coil as it rotates towards the microtubule minus-end. While many previous investigations have focused on the mechanisms of homodimeric kinesins, this work presents the first comprehensive study of the powerstroke of a heterodimeric kinesin. The stalk rotation shown here for Kar3Vik1 is highly reminiscent of that reported for the homodimeric kinesin-14 Ncd, emphasizing the conservation of a mechanism for minus-end directed motility.

## Introduction

The microtubule (MT)-associated motor, Kar3 [1] is the motor domain of a minus-end directed kinesin-14 with important roles in mitosis and karyogamy in Saccharomyces cerevisiae. In vivo Kar3 forms a heterodimer through specific coiled-coil interactions with either Vik1 [2] or Cik1 [3], two non-motor proteins that differentially control Kar3 localization and function [2], [4]. Vik1, and presumably Cik1, exhibit a tertiary folding pattern that is surprisingly similar to a kinesin motor domain but lacks an active site for ATP hydrolysis [5]. Additionally, Vik1 has been reported to bind tightly to MTs and interact directly with the MT during Kar3Vik1’s motile cycle [5], [6]. When Kar3 contacts the microtubule, release of ADP from Kar3 transitions the motor to a single-head bound state. Subsequent uptake of ATP triggers a rotation of Kar3Vik1’s coiled-coil towards the minus-end of the microtubule [6]. However, very little is understood about the nucleotide-free and ATP states where only one of the head domains is bound to the MT. Current models predict binding of Kar3 to the MT during these states but no direct evidence for this exists.

Despite being a heterodimer, the domain organization of Kar3Vik1 resembles that reported for Ncd, a homodimeric kinesin-14 required for proper chromosome segregation in Drosophila melanogaster [2], [7]. Kar3 and Vik1 each have globular domains at their C-termini that join together through a short coiled-coil neck. The X-ray crystal structure of Kar3Vik1 shows striking structural similarity to the asymmetric structures of Ncd, and similar to Ncd, there appears to be interaction between residues in the neck and in the C-terminal globular domains [6], [8], [9]. The neck extends directly into a longer coiled-coil stalk, which ends with two N-terminal globular domains. The N-terminal domains are basic, proline-rich regions that also bind to MTs, but in a non-ATP dependent manner [1]. This allows Kar3Vik1 to perform its function as a MT crosslinker to focus MTs at the spindle poles during mitosis [2].

Kar3Vik1 and other kinesin-14s are minus-end directed, non-processive motors that take only one step on the MT before detaching and preparing for a subsequent step. As has been shown with Ncd, it is likely that multiple motors are needed to generate movement of a MT [10]. This is in strong contrast to highly processive, plus-end directed kinesins such as kinesin-1 where MT movement can be achieved by a single functional unit [11], [12]. As individual Kar3Vik1 motors lack processivity, it is likely that they work in a coordinated manner with other Kar3Vik1 motors particularly in their role of focusing parallel MTs at the spindle poles and crosslinking MTs to provide stability to the mitotic spindle [2]. Indeed, Kar3Vik1 has been shown by immunofluorescence and cryo-electron microscopy (cryo-EM) to bind cooperatively to MTs in the presence of AMPPNP [5], [13]. In the presence of AMPPNP only Kar3 is predicted to bind to the microtubule surface. Hence, this suggests that cooperative binding is regulated by tight microtubule binding of Kar3 but not Vik1. Interestingly, immunofluorescence of the monomeric C-terminal motor head domain of Kar3 (Kar3MD) bound to MTs prepared in vitro, revealed that Kar3MD binds stochastically to MTs [14]. Here we use unidirectional surface shadowing to visualize the stochastic binding of individual Kar3MDs to MTs in order to illustrate how this differs from Kar3Vik1’s MT binding pattern in the nucleotide-free and ATP states.

To date, numerous structural studies have been conducted by cryo-EM 3-D analysis of the kinesin-microtubule interaction in the strong binding states, typically the nucleotide-free state (generated using the ATP- and ADP-hydrolyzing enzyme, apyrase), and the ATP state (mimicked with the non-hydrolyzable ATP analog, AMPPNP). The consensus of this work is that the individual kinesin head domains across different kinesin families bind in the same orientation to the same site on the MT [15], [16], [17], [18], [19]. However, homodimeric kinesin constructs from different families may have remarkably different MT interacting properties. For example, homodimeric kinesin-1 and kinesin-5 constructs bind to the MT with both motor head domains simultaneously to two consecutive tubulin dimers along the same protofilament [17], [20], [21]. The dimeric kinesin-6 construct of Zen4, a component of the centralspindlin complex [22] has mostly both heads bound, but there is less order in this dimeric binding configuration that may also reach across protofilaments [23]. In contrast, homodimeric Ncd has been shown to bind to the MT with only one of its motor heads, with the second head remaining detached [24].

A current study of Kar3Vik1’s mechanism of motion demonstrated that, like Ncd, only one head is bound to the microtubule in the nucleotide-free and AMPPNP states when observed by electron microscopy, though there is strong evidence by solution studies of a transient intermediate following MT collision where both heads are bound to the MT and Kar3 is nucleotide-free [6]. However, to date it has been impossible to distinguish whether it is Kar3 or Vik1 that is bound to the MT in the single-head bound states. Additionally, it is possible that a different head of the heterodimer is bound to the MT at distinct points in the motile cycle, especially given Vik1’s reported ability to bind tightly to MTs [5]. The possibility of Vik1 making initial contact with the microtubule surface while Kar3 has ADP in its active site has been discussed in detail by Allingham et al. [5] and Rank et al. [6]. As our cryo-EM work does not add to this model we focus on the three strong MT-binding states of Kar3Vik1.

Here we have employed cryo-EM and helical 3-D analysis of MTs decorated with a heterodimeric Kar3Vik1 [6] to analyze the relevant structural changes that occur within the heterodimer in response to changes in nucleotide state. With these methods we were able to directly visualize the powerstroke responsible for generating Kar3Vik1’s retrograde motility. Due to the similarity in size, shape and density of the C-terminal heads of Kar3 and Vik1, we could not unambiguously distinguish between these two domains from our cryo-EM data. Therefore, we designed a “cys-light” Kar3Vik1 construct with only one cysteine located on the C-terminal motor homology domain of Vik1. By labeling this cysteine residue specifically with a 1.4 nm maleimide-Nanogold® particle, we could show that for the nucleotide-free, ATP, and ADP+Pi states Kar3 maintains contact with the MT while Vik1 remains tethered. Additionally, analysis of the ADP+Pi state suggests that the coiled-coil does not revert to the pre-powerstroke position until after Kar3Vik1 dissociates from the MT. For a more detailed molecular interpretation of the structural changes that occur during Kar3Vik1’s motility cycle, we docked the X-ray crystal structure of Kar3Vik1 into our density maps (see also Rank et al., 2012 [6]. Our results reveal that Kar3Vik1 uses a powerstroke reminiscent of that described for Ncd, highlighting the conservation of a mechanism of movement among homo- and heterodimeric kinesin-14 motors.

## Results

### A Monomeric Kar3MD Construct Binds Stochastically to the MT Lattice While Dimeric Kar3Vik1 Binds Cooperatively

**Figure 1:**
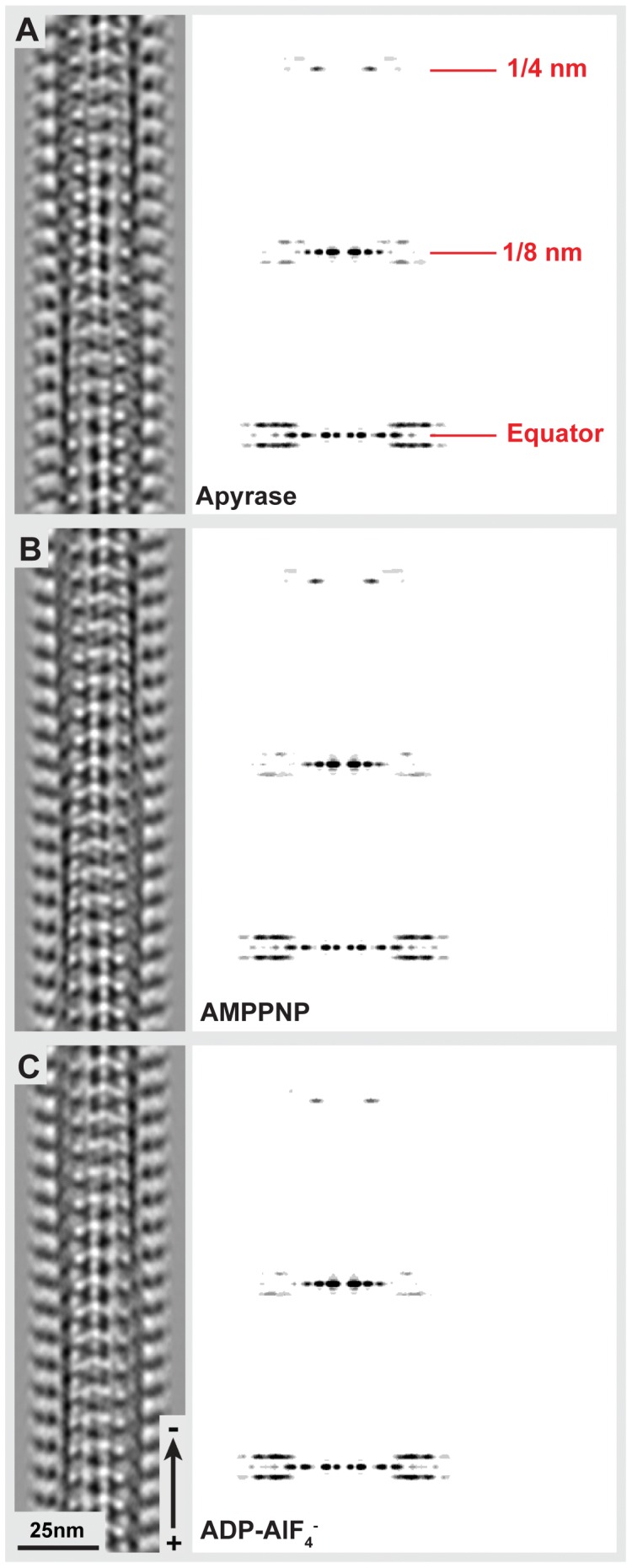
Kar3Vik1 binds to MTs in a cooperative manner. (**A**) High resolution heavy metal shadowing of Kar3MD monomers shows individual Kar3MDs binding to MTs in a stochastic (non-cooperative) manner. (**B**) Conversely, Kar3Vik1 binds to MTs in a cooperative fashion, which is demonstrated by one MT being completely decorated by Kar3Vik1 (right), while an adjacent MT is completely free of motor decoration (left). (**C**) A single MT showing one protofilament that is fully decorated by Kar3Vik1 while other protofilaments remain undecorated, illustrating that the cooperativity occurs in the axial direction along the MT. Cooperative decoration is strongly apparent in the nucleotide-free state (shown here), but is also seen in the AMPPNP [13] and ADP-AlF4- states.

A monomeric motor domain construct (Kar3MD; L383–K729) was incubated with MTs in the presence of the non-hydrolyzable ATP analog AMPPNP under sub-stoichiometric conditions (fewer Kar3MD domains than available binding sites on tubulin). Stochastic binding of Kar3MD to MTs has been reported previously by immunofluorescence (see [5], [14]), but here we employed electron microscopy to visualize individual Kar3MDs bound to the MT lattice. Kar3MD-MT complexes were examined by unidirectional shadowing (e.g. see [25]) which strongly emphasizes surface features. The resulting so-called shadowgraphs clearly reinforce that Kar3MD binds stochastically to the MT lattice where a Kar3MD does not bind preferentially to a site adjacent to another bound Kar3MD (Fig. 1A). Hence, unlike with the dimer (see below) the binding property of the isolated Kar3 motor domain does not show any cooperativity.

In the presence of AMPPNP the stochastic binding of monomeric Kar3MD is notably distinct from the heterodimeric Kar3Vik1’s striking ability to decorate MTs in a cooperative manner [13]. The cooperative mechanism described here occurs at a very small scale, confined so far to kinesin-14s and should not be confused with the long-range cooperativity observed by light-microscopy that seems to be a common effect observed on multiple kinesin families (e.g. see: [26]). By further examining additional nucleotide states for Kar3Vik1, we find that the cooperative binding effect is also strongly apparent in the nucleotide-free (Fig. 1B and C) and ADP-AlF4- states (data not shown). The cooperative decoration by Kar3Vik1 is seen in cryo-EM images showing MTs completely decorated by Kar3Vik1 while other MTs in close proximity remained undecorated by the motor (Fig. 1B). Furthermore, many MTs show partial, or even no decoration while some protofilaments are fully decorated by Kar3Vik1 (Fig. 1C). This binding pattern indicates that the cooperativity does spread laterally, but proceeds first in the axial direction along the length of the MT. Due to the static nature of electron microscopy images we cannot unambiguously determine if this enhanced binding property extends preferably towards the minus- or the plus-end. Similar cooperative binding has been reported for Ncd [27], but cooperative binding in the axial direction has not been noted for kinesin-1 [20], kinesin-5 [21], or kinesin-6 homodimers [23] which exhibit a distinct “two-heads-down” binding pattern in both the nucleotide-free and AMPPNP states (see [21]). The striking difference between the Kar3MD and Kar3Vik1 results here suggests that either the coiled-coil joining Kar3 and Vik1, or the Vik1MHD itself effects the cooperative binding properties of Kar3Vik1.

### Kar3Vik1 Binds to MTs through Only One of its Globular Domains in the Nucleotide-free, ATP and ADP+Pi States

**Figure 2:**
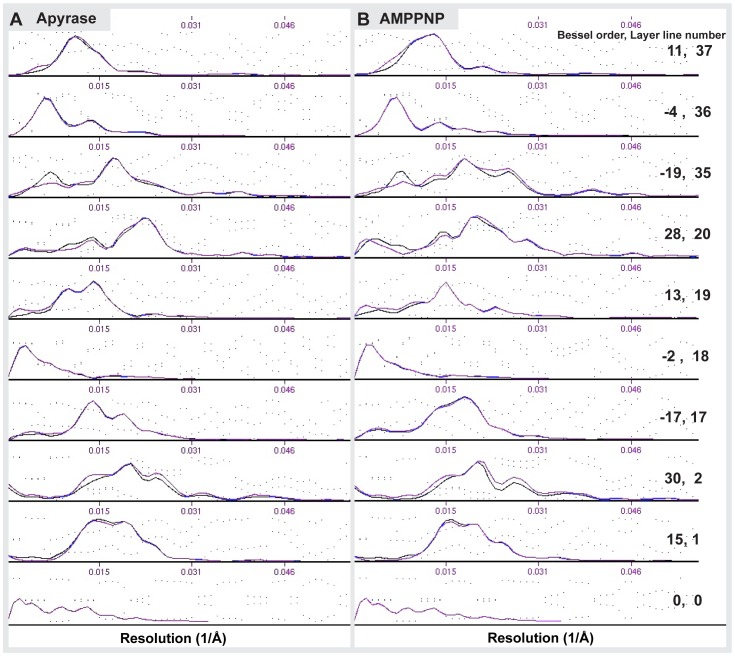
The MT binding configuration of Kar3Vik1 in different nucleotide states. Fourier-filtered images (top row) and corresponding Fourier transforms (bottom row) of Kar3Vik1 trapped on the MT at key points during its nucleotide hydrolysis cycle. (**A**) In the presence of Kar3Vik1-ADP, MTs appear predominantly undecorated. (**B**) In the nucleotide-free state, achieved by incubating Kar3Vik1 with apyrase, MTs are fully decorated and clearly show Kar3Vik1 binding to the MT with only one of its globular domains, with the second domain detached and extending away from the MT. Similarly, Kar3Vik1-AMPPNP, trapped in the ATP state (**C**), and Kar3Vik1-ADP-AlF4-, mimicking the ADP+Pi state (**D**) bind to the MT in a one-head-down, one-head-up configuration. Dotted red circles highlight the difference in the binding configurations of the nucleotide-free and AMPPNP states. The ADP- AlF4- state appears similar to the AMPPNP state configuration. The structural differences and similarities between the nucleotide states are shown in more detail in Fig. 3. In these images, it is not possible to know whether it is Kar3 or Vik1 in contact with the MT though this is addressed in Fig. 4.

Previous work has shown that in the presence of AMPPNP, only one of the globular domains of Kar3Vik1 is in contact with the MT surface [13]. Here we confirm this binding configuration for the AMPPNP state (Fig. 2C) and show that Kar3Vik1 also binds in a one-head-down, one-head-up fashion in the nucleotide-free (Fig. 2B) and ADP-AlF4- (Fig. 2D) states. From Fourier-filtered 2D-projections (Fig. 2), it is clear that in each of these nucleotide states, one Kar3Vik1 heterodimer binds per αβ-tubulin dimer. However, due to the similar size and shape of the Kar3 and Vik1 globular domains, it is impossible to know which component of the heterodimer is in contact with the MT in each of these nucleotide states. Hence, we addressed this issue below with the help of site-directed labeling.

### In the Nucleotide-free State, Kar3Vik1’s Coiled-coil Points Toward the MT Plus-end

**Figure 3:**
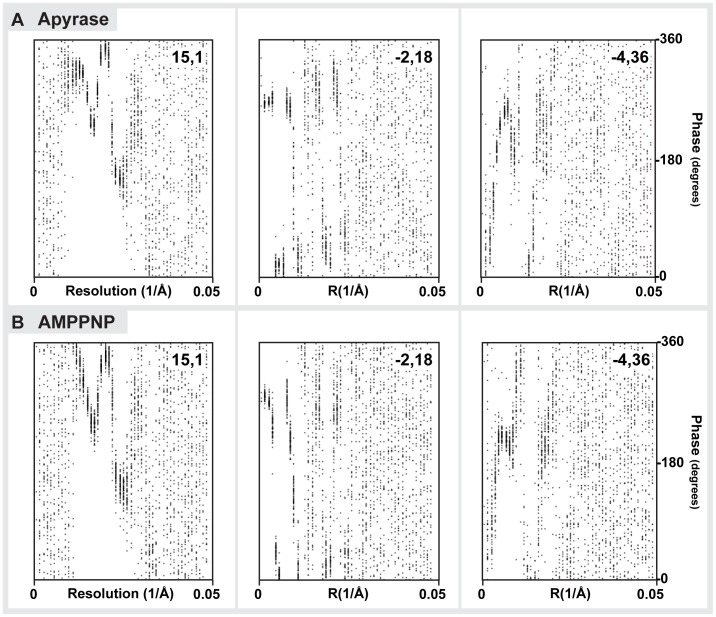
Cryo-EM and helical reconstruction reveals a crucial part of the Kar3Vik1 powerstroke. Helical reconstructions of Kar3Vik1-decorated MTs in the nucleotide-free (**A**), AMPPNP (**B**), and ADP-AlF4- (**C**) states. The left hand column shows 0.38 nm cross-sectional views through the 3-D helical averages with the MT plus-end pointing toward the reader. The right hand column shows surface renderings of the helical averages with the MT plus-end pointing toward the bottom of the page. (**A**) In the nucleotide-free state, Kar3Vik1's coiled-coil stalk can be seen pointing toward the MT plus-end. (**B**) The AMPPNP state reveals a large structural change in the outer domain that rotates the coiled-coil stalk ∼75° to position it toward the MT minus-end. This stalk rotation is likely to have a crucial role in facilitating Kar3Vik1’s minus-end directed motility. Key structural differences between the nucleotide-free and ATP states are highlighted with colored circles. (**C**) Cryo-EM helical reconstruction of Kar3Vik1 complexed to MTs in the ADP-Pi state shows that when incubated with ADP-AlF4-, Kar3Vik1 adopts a MT-binding configuration very similar to that of the AMPPNP state with the coiled-coil stalk pointing toward the MT minus-end. Color key: tubulin - turquoise, Kar3 - gray, Vik1 - yellow, GCN4 coiled-coil - pale blue.

Kar3Vik1-MT complexes in the nucleotide-free state were analyzed by helical reconstruction to determine the MT-bound structure of Kar3Vik1 that occurs when Kar3 contacts the MT and releases nucleotide. The 3D map from helical averaging of Kar3Vik1 bound to MTs in the nucleotide-free state (EMDB accession: EMD-5416) shows the two head domains of Kar3 and Vik1 stacked on the MT with only one head interacting with tubulin (Fig. 3A). A distinct elongated density can be seen extending between the two heads. The location and shape clearly indicate that this density corresponds to Kar3Vik1’s coiled-coil neck and stalk. In the nucleotide-free state, the stalk points toward the plus-end of the MT (Fig. 3A). Optical diffraction patterns and phase-amplitude plots of the helical 3-D maps are shown in Figures S1, S2 and S3. The resolution of the 3-D map is estimated to be approximately 2.2 nm based on Fourier-Shell correlation (Fig. S4).

### Binding of ATP to Kar3 Causes a Rotation of Kar3Vik1’s Stalk Toward the MT Minus-end

The helical reconstruction density map of Kar3Vik1-AMPPNP bound to MTs (EMDB accession: EMD-5417) looks strikingly different from that of Kar3Vik1 bound in the nucleotide-free state (Fig. 3B). At a resolution of 2.2 nm as estimated by Fourier Shell correlation (Fig. S4), the microtubule and MT-bound Kar3Vik1 domain remain almost identical. However, the position of the tethered domain is quite different, as is the density corresponding to the coiled-coil stalk that once again is well resolved, but now points toward the minus-end of the MT (Fig. 3B). The stalk appears to rotate approximately 75° upon uptake of AMPPNP. In addition to the rotation of the stalk, the outer domain also undergoes a significant rotation as highlighted by the red- and black-boxed regions (Fig. 3A and B insets). The isosurface representations in Fig. 3 may be misleading with regard to the true connections of the stalk to the motor. One has to keep in mind that iso-surfaces do not discriminate between a true molecular connection and a close contact.

### The ADP+Pi State Strongly Resembles the ATP State

ADP-AlF4- [28] is believed to mimic an ADP+Pi like transition state in kinesins, as demonstrated for kinesin-1 and Ncd (e.g. see [29], [30], [31]), as well as myosins [32]. Here we used ADP-AlF4- to trap Kar3Vik1 in a state thought to represent Kar3Vik1’s structure at the transition state for ATP hydrolysis, before the release of Pi from the active site. Our 3-D maps produced by helical reconstruction of Kar3Vik1 ADP-AlF4- bound to MTs strongly resemble that of the Kar3Vik1-AMPPNP MT-bound state (Fig. 3C). The resemblance of the maps of the AMPPNP and ADP-AlF4- binding states suggests that Kar3Vik1 does not use the hydrolysis of ATP to ‘reset’ the position of its stalk to a pre-powerstroke position (the position seen in the ADP-bound [6] and nucleotide-free states (Fig. 3A)). Rather, ATP hydrolysis may convert the motor to a weak MT-binding state and the stalk returns to its original position only after Kar3Vik1 detaches from the MT.

### Kar3 is the Domain in Contact with the MT in the Nucleotide-free and ATP States

**Figure 4:**
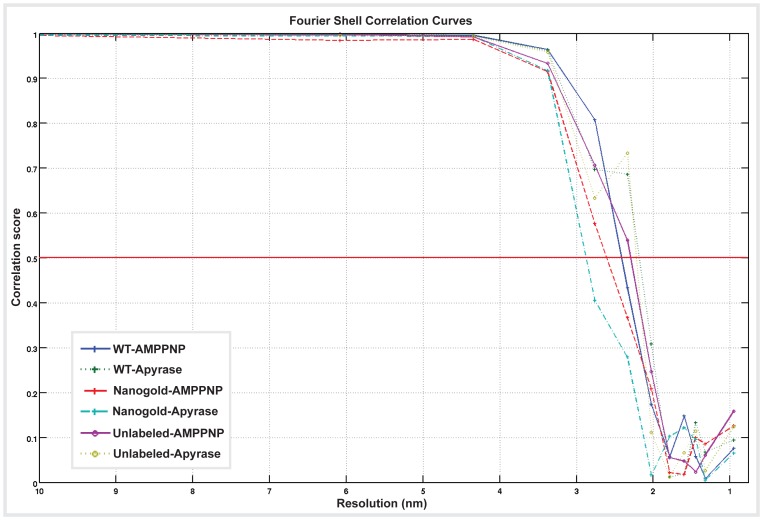
Cryo-EM helical reconstruction and comparison of unlabeled and Nanogold®-labeled Kar3CFVik1C536C-MT complexes. (**A & B**) 0.38 nm cross-sections through helical averages of MTs decorated with GCN4-Kar3CFVik1C536C that has not been labeled with Nanogold®, in the nucleotide-free (A) and AMPPNP (**B**) states. These maps appear indistinguishable from those of wild-type GCN4-Kar3Vik1 (Fig. 3). (**C & D**) 0.38 nm cross-sections through helical averages of MTs decorated with Nanogold®-labeled GCN4-Kar3CFVik1C536C. An additional density (orange circle) corresponding to the Nanogold® is clearly seen protruding from the outer domain in both the nucleotide-free (**C**) and AMPPNP states (**D**). (**E**) Difference map calculated by subtracting the unlabeled map in A from the Nanogold®-labeled map in **C**. Differences that are statistically significant for P<0.001 are shown in color. The difference map is overlaid on the helical average from **A** to show that the significant differences correspond to the position of the Nanogold® providing evidence that Vik1 is the outer domain and Kar3 is in contact with the MT. (**F**) Difference map obtained by subtracting the average in **B** from the average in **D**, overlaid on the average from **B**, shows similar results for the AMPPNP state.

Given the strong structural similarities between Kar3 and Vik1 at the resolution of the helical 3-D maps, and Vik1’s reported ability to bind to MTs [5] it was necessary to distinguish between Kar3 and Vik1 to properly identify the location of the two domains in both the nucleotide-free and AMPPNP states. To this end we employed site-directed Nanogold®-labeling that provides a high-electron diffracting particle that can be specifically linked to exposed cysteines via a maleimide linker. This allowed us to unambiguously determine which globular head domain of the heterodimer (either Kar3 or Vik1) is in contact with the MT and which domain appears to aid the coiled-coil stalk rotation. A single-cysteine Kar3Vik1 construct, GCN4-Kar3CFVik1C536C was engineered that could be labeled specifically with a maleimide-Nanogold® tag at residue 536 on Vik1. Helical averages of unlabeled GCN4-Kar3CFVik1C536C complexed to MTs in the nucleotide-free and AMPPNP states were reconstructed as controls and for difference mapping with the labeled constructs. The 3-D maps of unlabeled complexes were indistinguishable from those of WT GCN4-Kar3Vik1 (Fig. 4A and B). Difference maps revealed that unlabeled and labeled maps were identical with the exception of the density due to the Nanogold® particle which could be easily located as it produced a large density due to its strong electron scattering power. Taken together these data indicate that neither the replaced residues in the single-cysteine construct nor the added gold label had any adverse effects on motor configuration when bound to MTs as seen by cryo-EM.

**Figure 5:**
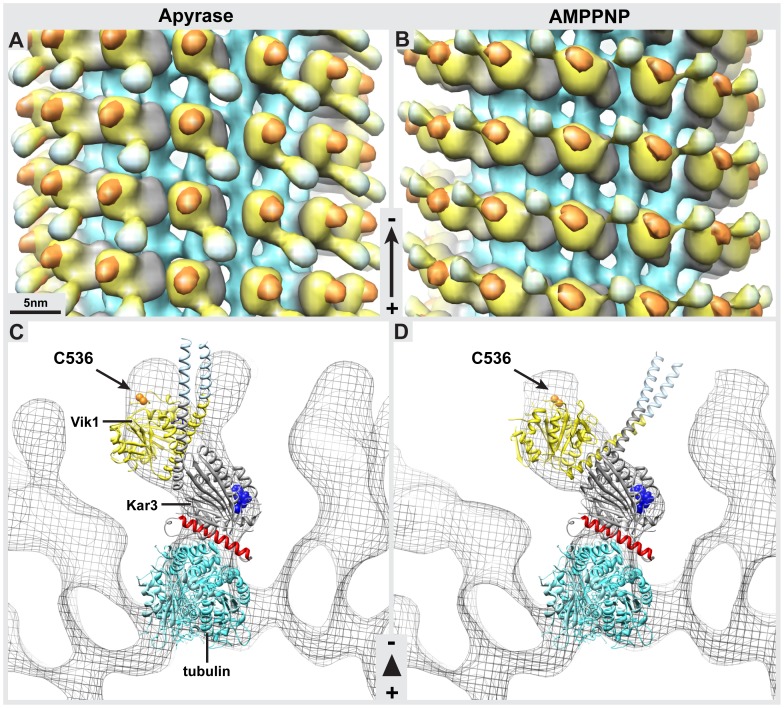
Nanogold®-labeling shows Kar3 is in contact with the MT in the nucleotide-free and AMPPNP states. Longitudinal views of helical averages of Nanogold®-labeled GCN4-Kar3CFVik1C536C in the nucleotide free (**A**) and AMPPNP (**B**) states. The density corresponding to the gold label is colored orange. (**C & D**) The X-ray crystal structures of tubulin (PDB accession: 1JFF; [33]) and Kar3Vik1 (PDB accession: 4ETP; [6]) docked into the scaffolds of Nanogold®-labeled GCN4-Kar3CFVik1C536C helical averages in the nucleotide-free (**C**) and AMPPNP states (**D**). Vik1 C536, the residue that was labeled with Nanogold® is colored in orange showing close agreement with the location of the Nanogold® in the helical reconstructions. The ADP molecule bound in the Kar3 active site on the crystal structure is shown in blue and helix α4 of Kar3 is shown in red. Color key: tubulin - turquoise, Kar3 - gray, Vik1 - yellow, GCN4 coiled-coil (isosurface) or SHD coiled-coil (crystal structure) - pale blue, Nanogold® - orange.

Three-dimensional maps obtained by helical reconstruction of Nanogold®-labeled GCN4-Kar3CFVik1C536C bound to MTs in the nucleotide-free state showed a distinct additional density on the head extending away from the MT (Fig. 4C and 5A). Similarly, in the AMPPNP state, a clear extra density protruding from the outer head of the heterodimer was detected (Fig. 4D and 5B). Statistical analysis of the difference maps, carried out to compare helical reconstructions of unlabeled GCN4-Kar3CFVik1C536C and Nanogold®-labeled GCN4-Kar3CFVik1C536C, showed that the additional density difference in both states is statistically significant (P<0.001) (Fig. 4E and F).

We have recently published a detailed docking of the Kar3Vik1 heterodimer X-ray crystal structure (PDB accession: 4ETP) into 3D maps of wild-type GCN4-Kar3Vik1-microtubule complexes obtained from helical reconstruction [6]. When the GCN4-Kar3Vik1 maps from these docking experiments are replaced with those of Nanogold®-labeled GCN4-Kar3CFVik1C536C, the single cysteine correlates well with the difference densities and hence corresponds perfectly to the position of the Nanogold® label with high probability (Fig. 5C and D). These results confirm that in the nucleotide-free and AMPPNP states, Kar3 is in contact with the MT, while Vik1 rotates together with the motor’s coiled-coil stalk.

## Discussion

This work represents a detailed structural and functional study on the interaction between MTs and the heterodimeric kinesin-14 Kar3Vik1, and as such complements our initial tomographic 3-D investigation on Kar3Vik1-MT complexes [13], as well as the report on the Kar3Vik1 X-ray crystal structure [6]. Using cryo-EM and helical 3-D image reconstruction we have shown that in the nucleotide-free, ATP, and ADP+Pi states, Kar3Vik1 contacts MTs in a one-head-down, one-head-up binding configuration. This is not an enforced state due to overcrowding, but, as previously seen with dimeric Ncd [27] is the natural microtubule-binding structure, and persists on both, fully and partially decorated microtubules (see also figure 3 of ref [13]. It might be the source for the strong cooperative binding property that is typical for Ncd as well [27]. Also, this binding pattern is very different from all anterograde kinesins studied so far (e.g. see [17] for kinesin-1, [21] for kinesin-5, and [23] for kinesin-6). We also demonstrate that Kar3Vik1 binds to MTs in a highly cooperative fashion, very different from Kar3MD alone which binds stochastically to the MT lattice, but similar to the cooperative decoration seen by Ncd [27]. This is a significant finding that implicates the role of the heterodimeric coiled-coil and/or Vik1MHD in the cooperative binding behavior of Kar3Vik1. The 3-D reconstruction approach used here was not suitable to analyze the Kar3Vik1-MT binding configuration in the presence of ADP as only low-affinity, stochastic binding of Kar3Vik1 to the MT lattice is observed [6]. Hence, we focused on the structural details of the minus-end directed powerstroke of Kar3Vik1 as it occurs during three consecutive nucleotide states (nucleotide-free, ATP, and ADP+Pi) that induce a strong MT binding affinity and stoichiometric MT decoration that is required for helical 3-D analysis.

### ATP Uptake in Kar3Vik1 Produces a Minus-end Directed Rotation of its Stalk Domain

Our cryo-EM based helical 3-D reconstructions of Kar3Vik1-MT complexes revealed that Kar3Vik1 undergoes a large structural change upon uptake of ATP into the empty nucleotide pocket of Kar3. The result is a swing of Kar3Vik1’s coiled-coil stalk that is supported by a large rearrangement of the Vik1 domain. These rearrangements result in a ∼75 degree stalk rotation from its plus-end directed position in the nucleotide-free state, toward the MT minus-end upon uptake of ATP. Through the use of a maleimide-Nanogold® label, we provided direct evidence that Kar3 is the domain in contact with the MT for the duration of the powerstroke, while Vik1 is detached from the microtubule, but remains associated with the coiled-coil, perhaps facilitating rotation of the stalk.

**Figure 6:**
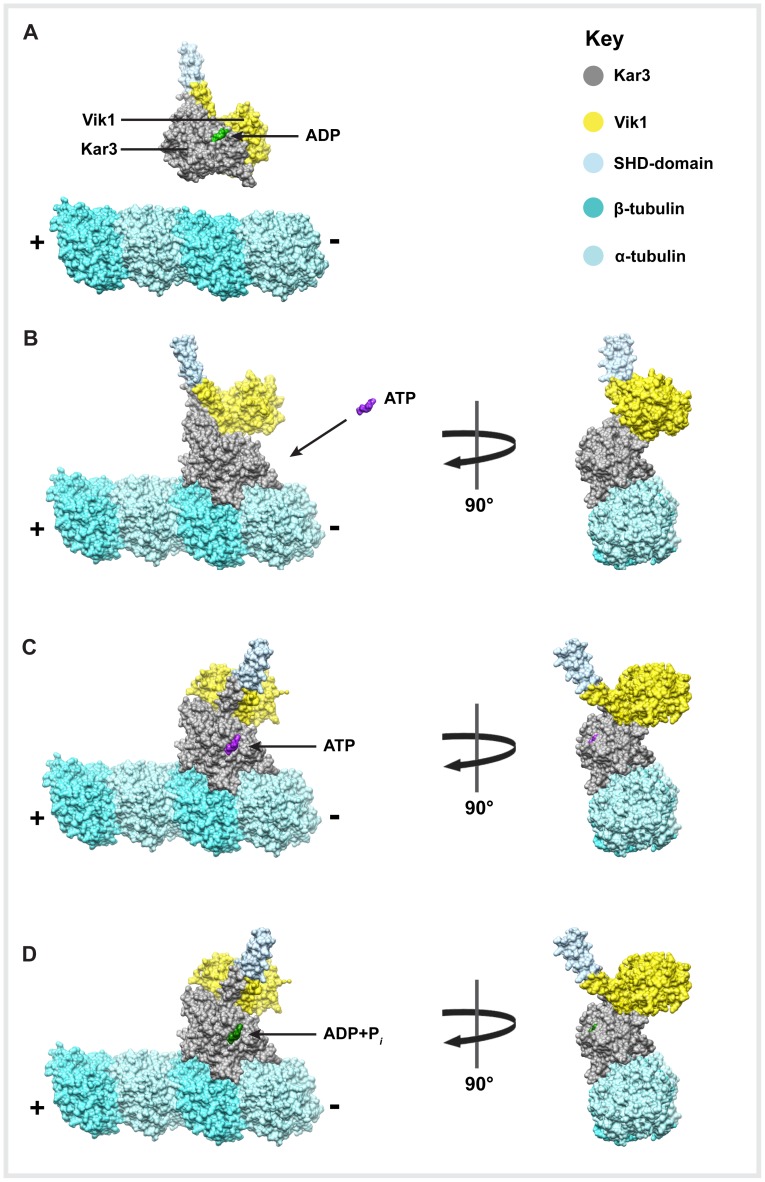
Kar3Vik1 uses a stalk rotation for minus-end directed movement. This model is proposed based on the structural changes seen in helical reconstructions (Fig. 3) and X-ray crystal structure docking into the density maps obtained from helical averaging (αβ-tubulin, PDB accession: 1JFF, [33]; Kar3Vik1, PDB accession: 4ETP, [6]). (**A**) When Kar3Vik1 is not bound to MTs, ADP is present in Kar3’s nucleotide-binding site. (**B**) Kar3 makes contact with a MT, which induces ADP release from the active site, but does not cause large-scale structural changes in Kar3Vik1 based on the crystal structure. (**C**) ATP is subsequently taken up into Kar3’s active site which results in a ∼75° rotation of Kar3Vik1’s coiled-coil that displaces the motor’s cargo MT a short distance toward the MT minus-end. (**D**) ATP is then hydrolyzed, which does not lead to a large-scale structural rearrangement, but does reduce the affinity of Kar3 for the MT. Kar3Vik1 can then detach from the MT and the coiled-coil stalk returns to its original position in preparation for a subsequent step.

To further analyze the Kar3Vik1 stalk rotation in molecular detail we docked the X-ray crystal structure of Kar3Vik1 (PDB accession: 4ETP, [6]) and the αβ-tubulin dimer (PDB accession: 1JFF, [33]) into the 3-D density maps obtained from helical reconstruction. The details about the docking procedure are described below (Mat. & Meth.). Despite truncating our maps to 2.2 nm, the stalk-related density was well visible in all wild-type GCN4-Kar3Vik1 3-D maps (Fig. 3). We accommodated the structural changes by moving the three major domains: Kar3MD (G385–T723), Vik1MHD (G373–Q638), and the coiled-coil stalk (Kar3 A332-R384, Vik1 A320-K372) as rigid bodies with hinges to match the cryo-EM densities. A detailed docking experiment between our non-nucleotide map and the ADP-crystal structure has been described in Rank et al., 2012 [6]. Here, we have used the results of our docking experiments [6] and Nanogold-labeling (this paper) to reveal a mechanistic model illustrating Kar3Vik1’s powerstroke used for minus-end directed movement (Fig. 6). When Kar3Vik1 is not bound to MTs, ADP is complexed in the active site of Kar3, which is widely accepted as the solution state for kinesin motors in general. When Kar3 makes contact with a MT, ADP release from the Kar3 active site is stimulated [5], [34]. Due to the almost perfect fit of the overall 3-D configuration of the Kar3Vik1-ADP X-ray crystal structure into the nucleotide-free state cryo-EM map (see Fig. 5C in this paper and Fig. 4A and B in Rank et al., 2012 [6]), it appears that very little structural rearrangements occur relative to the crystal structure of Kar3Vik1. The fit could be improved by a small angular rotation of the coiled-coil stalk (see Fig. 5C), which may indicate a rearrangement that occurs upon microtubule binding and/or loss of nucleotide from the Kar3 active site, or may simply be an artifact that was induced by the crystal packing. The nucleotide-free state clearly shows Kar3Vik1’s coiled-coil stalk pointing toward the MT plus-end. When ATP is taken up into Kar3’s active site, it causes a ∼75° rotation of Kar3Vik1’s stalk that is likely the key-structural change that pushes Kar3Vik1’s cargo, in this case another MT, towards the minus-end. This step thus seems to represent the actual ‘powerstroke’ of Kar3Vik1 and the two domains change position in a way that the stalk cannot flip backwards again. ATP is then hydrolyzed and transforms Kar3Vik1 into an ADP+Pi state that remains in contact with the MT surface through the Kar3MD only. According to our 3-D data, unlike ATP uptake, ATP hydrolysis does not induce large structural rearrangements, but instead prepares the motor domain to detach from the MT, either before or after release of Pi from the active site. Once detached from the MT, the coiled-coil stalk resets to its original position and readies the motor for its next step, a mechanism that characterizes a non-processive motor.

While Vik1 and the coiled-coil stalk undergo a rotation upon ATP binding, Kar3 appears to maintain its position on the MT and does not, or only slightly, rotates with the rest of the complex (Figs. 3 and 5). This result is consistent with the findings of Hirose et al. [18] whose cryo-EM work with the monomeric Kar3MD did not show a rotation of Kar3 on the MT in response to changes in nucleotide state. Hence, the binding of ATP to the active site triggers rearrangements that are transmitted through the molecule to initiate the powerstroke. The resolution in our cryo-EM maps is not high enough to be able to clearly identify the structural elements responsible for this. Previously suggested models have proposed that changes in helix α4 (highlighted in red in Fig. 5) and a subsequent shift of the central β-sheet are likely to be important for communicating the presence of nucleotide to the rest of the complex, causing the changes necessary for detaching the neck from the Kar3 core to facilitate the minus-end directed stroke [18].

### Kar3Vik1’s Powerstroke Resembles that of Homodimeric Ncd

The distinct nucleotide-induced 3-D structures of Kar3Vik1 bound to MTs, as well as the cooperative MT binding pattern of Kar3Vik1, are both highly reminiscent of the homodimeric kinesin-14 Ncd [24], [27], [31]. This comes as a slight surprise since Ncd features two identical motor heads rather than a single motor domain (Kar3) and a motor homology domain (Vik1). Cryo-EM and helical reconstruction of Ncd-MT complexes have shown that the Ncd stalk points to the MT plus-end in the nucleotide-free state, and undergoes a ∼70° rotation toward the minus-end upon uptake of AMPPNP. The Ncd-ADP-AlF4- binding state also closely resembles the AMPPNP state and is similar to the Kar3Vik1-ADP-AlF4- maps presented here [31]. These results have led to the proposal that Ncd uses a ‘lever-arm’ rotation to power its retrograde movement. This lever-arm model has been well supported by evidence demonstrating that the MT-gliding velocity of a number of Ncd truncation or extension mutants is proportional to the length of the coiled-coil stalk [31].

On a closer look, the similarities between the Kar3Vik1 and Ncd powerstroke mechanisms may be attributed to very similar structural features. At the sequence level, key amino acids reported to be critical for Ncd’s minus-end directed motility are also conserved in Kar3. Notably, a point mutation, N340K, close to the base of Ncd’s coiled-coil neck has been shown to severely interfere with the minus-end directionality of Ncd [35]. This residue is conserved in Kar3 (N378) and indeed a Kar3-N378K mutant is a more powerful suppressor of otherwise lethal kinesin-5 knockouts than a complete Kar3 deletion [36]. This is a strong indication that Kar3-N378K may substitute for some of the outward force otherwise provided by plus-end directed kinesin-5 motors. In addition, the mutation K460A in Ncd results in a three-fold decrease in Ncd’s MT gliding velocity, and an Ncd triple mutant Q420A/S421A/Y426A glides MTs ten times slower than wild-type Ncd [37]. All four of these residues are conserved in Kar3 and are found at comparable locations in the 3-D structure (compare [37] with [6]).

Furthermore, the residues at the base of the neck (G347 in Ncd, G385 in Kar3 and G373 in Vik1) are highly conserved across all kinesin-14s [38]. At Ncd-G347, the coiled-coil ends and makes a sharp turn to the first residue of the globular head domain. G347 is thus believed to be the pivot point for the stalk rotation that occurs upon uptake of ATP. Importantly, many of the residues mentioned here are conserved only among the C-terminal kinesin-14′s, which further indicates their fundamental role in minus-end directed stepping and distinguishes it from plus-end directed movement of kinesins.

The work presented here highlights the conservation of a lever arm rotation as the underlying mechanism of movement among minus-end directed kinesins (kinesin-14 family), whether homo- or heterodimeric in nature. Nevertheless, despite their similarities, Kar3Vik1 and Ncd have a striking difference in that Ncd is a homodimer of two motor heads, while Kar3Vik1 has one motor domain and a motor homology domain. Motility experiments with a one-headed Ncd heterodimer revealed that the one-headed motor was able to glide microtubules at a velocity comparable to wild-type homodimeric Ncd [31]. This result demonstrated the importance of an intact coiled-coil stalk for movement but implied that the second Ncd head was not necessary for proper motility. An analogous one-headed Kar3Vik1 construct (that lacks the Vik1 C-terminal motor homology domain) has not yet been tested, but it would be very interesting to see how deletion of the Vik1MHD would affect motility. Though Vik1 plays a functional role in Kar3Vik1 promoted movement, the results presented here demonstrate that Vik1 rotates with the coiled-coil during Kar3Vik1’s powerstroke rather than remaining bound to the MT.

## Experimental Procedures

### MT Polymerization

MTs were polymerized in vitro from 45 µM bovine brain tubulin (Cytoskeleton, Inc., Denver, CO) with BRB80 (80 mM PIPES, pH 6.8, 1 mM MgCl2, 1 mM EGTA) in the presence of 1 mM GTP, 10 µM paclitaxel (Sigma, St. Louis, MO) and 7.5% (v/v) DMSO for 30 min at 35°C, and allowed to stabilize overnight at room temperature. MTs were always used within 24 hours after polymerization at 35°C.

### Unidirectional Heavy-metal Shadowing of Kar3MD-MT Complexes

Kar3MD at 4.5 µM in ATPase buffer and 2 mM AMPPNP was added to polymerized MTs at a total tubulin dimer concentration of 3.75 µM and incubated for 90–120 s. Kar3MD-MT complexes were vitrified by plunging the grid into liquid ethane. Following plunge-freezing, grids were freeze-dried and unidirectionally shadowed with tantalum/tungsten as described [25].

### Protein Expression and Purification

The monomeric Kar3MD construct used here contains residues L383–K729 of wild-type (WT) Kar3, which corresponds to the entire head domain. Kar3MD was expressed and purified as described previously [39]. WT GCN4-Kar3Vik1 was expressed as reported previously [6]. WT GCN4-Kar3Vik1 is a truncated version of Kar3Vik1 containing residues K353–K729 of Kar3, and S341–T647 of Vik1 each fused to four heptads of GCN4 with the sequence SVKELEDKVEELLSKNYHLENEVARLKKLV to stabilize the dimer. Kar3 included a TEV protease cleavable N-terminal His tag (MGSSHHHHHHHHDYDIPTSENLYFQGASM) and Vik1 a TEV protease cleavable StrepII tag (MASWSHPQFEKENLYFQGAS) where residues removed by TEV are indicated in italics. The WT GCN4-Kar3Vik1 construct includes the complete C-terminal globular domains of Kar3 and Vik1 and two and a half heptads of the native coiled-coil stalk through which Kar3 and Vik1 heterodimerize. For Nanogold® labeling, a previously characterized GCN4-Kar3Vik1 construct with all cysteines removed that displays wild type activity [6], was mutated to add back a single native cysteine at position 536 in Vik1 (GCN4-Kar3CFVik1C536C) allowing for specific labeling of Vik1. GCN4-Kar3CFVik1C563C is identical to the WT GCN4-Kar3Vik1 construct except for the following mutations: Kar3 C391L, C469A, C517A, C566V, and C655V and Vik1 C377V, C436A, C596A, and C640A.

### Preparation of Frozen-hydrated Kar3Vik1-MT Complexes for cryo-EM

GCN4-Kar3Vik1-MT complexes were assembled directly on holey carbon C-flat grids (Protochips, Inc., Raleigh, NC) to prevent bundling of the MTs. Polymerized MTs were diluted to a total tubulin dimer concentration of 3.75 µM with BRB80 and 5 µl of diluted MTs were allowed to adsorb to a holey carbon grid for approximately 50 seconds. Excess liquid was blotted away and immediately 5 µl of GCN4-Kar3Vik1 with the appropriate nucleotide analog was added to the MTs for 90–120 seconds before blotting away excess liquid and rapidly plunging the grid into a cup of liquid ethane using a simple mechanical plunge-freezing device.

#### Nucleotide-free state

The nucleotide-free state was achieved by incubating GCN4-Kar3Vik1 with apyrase grade VII (Sigma, St. Louis, MO). GCN4-Kar3Vik1 was diluted to 4.5 µM with ATPase buffer (20 mM HEPES pH 7.2, 5 mM magnesium acetate, 50 mM potassium acetate, 0.1 mM EDTA, 0.1 mM EGTA, 1 mM DTT) and 16 U/ml apyrase, and incubated on ice for 30–45 minutes. A 5µl droplet of nucleotide-free GCN4-Kar3Vik1 was added to MTs adsorbed on a holey carbon grid and allowed to incubate for 90–120 seconds before blotting and plunging into liquid ethane as described above.

#### ATP state

GCN4-Kar3Vik1 was trapped in the ATP state with the non-hydrolysable ATP analog adenylyl imidodiphosphate tetralithium salt (AMPPNP) (Sigma, St. Louis, MO). GCN4-Kar3Vik1 at a final concentration of 8 µM in ATPase buffer was incubated with 2.2 mM AMPPNP on ice for 10–30 minutes. A 5µl droplet of GCN4-Kar3Vik1 complexed to AMPPNP was added to MTs adsorbed on a holey carbon grid and incubated for 90–120 seconds before blotting and plunging as described above.

#### ADP+Pi state

The transition state analog ADP-AlF4- was used to trap GCN4-Kar3Vik1 in a state thought to represent Kar3Vik1’s 3-D structure after hydrolysis of ATP, but before the release of Pi from the active site. ADP-AlF4- was prepared using ADP, AlCl3 and KF at final concentrations of 4 mM, 2 mM and 30 mM respectively in PME buffer (10 mM PIPES, 5 mM MgCl2, 1 mM EGTA) with 50 mM KCl (KF was prepared fresh in a plastic tube immediately before use). GCN4-Kar3Vik1 at a final concentration of 14 µM was added to the ADP-AlF4- solution and incubated for 2 min. Instead of assembling motor-MT complexes on the grid, MTs were added to the Kar3Vik1-ADP-AlF4- mixture at a final concentration of 3.5 µM, and incubated at room temperature for 6–15 minutes. A 5-µl droplet of the Kar3Vik1-ADP-AlF4- - MT mixture was applied to a grid for 60 seconds before blotting and plunge-freezing.

### Nanogold®-labeling of GCN4-Kar3CFVik1C356C

Maleimide-Nanogold® (Nanoprobes, Yaphank, NY) was added in a 2-fold molar excess to 3 nmol of GCN4-Kar3CFVik1C536C, and Nanogold® labeling of the single cysteine residue on Vik1 was allowed to proceed for 10 hours at 4 °C. After labeling, the GCN4-Kar3CFVik1C536C-Nanogold® mixture was applied to a Sephadex-G75 size exclusion column (GE Healthcare, Uppsala, Sweden) to separate the Nanogold®-labeled GCN4-Kar3CFVik1C536C from unbound Nanogold®. Nanogold®-labeled GCN4-Kar3CFVik1C536C was eluted from the column in PME buffer (10 mM PIPES pH 6.9, 5 mM MgCl2, 1 mM EGTA) with 100 mM KCl, 0.5 mM TCEP and 0.1 mM ATP. Labeling efficiency was shown to be 70–80% determined by separating Nanogold®-labeled Vik1C536C from unlabeled Vik1C536C by SDS-PAGE and comparing their relative band intensities on the gel using ImageJ (National Institutes of Health, Bethesda, MD).

### Cryo-EM Data Collection

Plunge-frozen samples were transferred under liquid nitrogen to a Gatan-626 cryo-holder (Gatan, Inc, Pleasanton, CA). Two-dimensional images of vitrified Kar3Vik1-decorated MTs were acquired on an FEI Tecnai F20 FEG transmission electron microscope (FEI-Company, Eindhoven, The Netherlands) operating at 200 kV. Single-frame images were taken at a nominal magnification of 29000x and a defocus of -2.5 µm with a total electron dose of 1500 electrons/nm2. Images were recorded without binning on a 4K×4K Gatan Ultrascan 895 CCD camera (Gatan, Inc, Pleasanton, CA). At this magnification, the resulting pixel size corresponds to 0.38 nm on the specimen.

### Helical Reconstruction of Kar3Vik1-MT Complexes

Cryo-EM CCD images were visually inspected for fully decorated MTs with fifteen protofilaments. Using IMOD [40], fifteen-protofilament/two-start helical MTs were extracted from the images and rotated to lie horizontally. Helical processing was carried out using PHOELIX as described previously [41]. In brief, each MT was computationally straightened and layer-line information was extracted. Layer-line data from a large number of MTs were shifted to a common phase origin using a reference and subsequently averaged. Three rounds of averaging were carried out with iterative improvement of the reference used to align the data. Helical averaging was always completed with more than one reference to ensure that final averages were not biased by the reference used. Only datasets with a phase residual of less than 20 relative to the reference were included in the final reconstruction. The final dataset was truncated to a maximum resolution of 2.2 nm which, at the imaging conditions used here marks the position of the first so-called Thon ring produced by the contrast transfer function (CTF: see also Fig. S1). 3-D density maps of the averages were visualized in IMOD. Surface rendering of the maps was carried out in UCSF Chimera [42]. Phase-amplitude plots of the merged average (Fig. S2) and the overlay of all individual datasets (Fig. S3) provide an objective measure of the data quality. The resolution of the maps is estimated by Fourier-Shell correlation to be around 2.2–2.5 nm (Fig. S4).

The number of individual datasets and approximate number of asymmetric units included in each of the helical reconstructions is shown in Table 1.

**Table 1:** Kar3Vik1 constructs and approximate number of asymmetric units included in cryo-EM helical averages.

Kar3Vik1 construct	No. of datasets	Approx. no. of asymmetric units
GCN4-Kar3Vik1– Nucleotide-free	52	42 000
GCN4-Kar3Vik1– AMPPNP	67	27 000
GCN4-Kar3Vik1– ADP-AlF_4_^-^	14	6 000
GCN4-Kar3_CF_Vik1_C536C_ – Nucleotide-free	59	37 500
GCN4-Kar3_CF_Vik1_C536C_ – AMPPNP	37	36 000
Nanogold®-GCN4-Kar3_CF_Vik1_C536C_ – Nucleotide-free	46	22 000
Nanogold®-GCN4-Kar3_CF_Vik1_C536C_ – AMPPNP	33	15 000

### T-test Based Difference Mapping to Reveal Significant Differences

The variance within datasets used for each helical reconstruction was determined by generating individual reconstructions from each of the near and far side datasets that were included in the final reconstruction. Each of the datasets from which individual reconstructions were created were all shifted to the same phase origin and their density scaled so that they could be compared to each other voxel by voxel. The standard deviation of the voxels at each corresponding position across all of the individual reconstructions was calculated. To calculate differences, the final helical average from one state was subtracted from the final average of a second state. The statistical significance of the difference at each voxel was determined using the standard deviation maps showing the internal differences for each of the two states. Differences were deemed ‘significant’ using a one-tailed t-test with a significance level of 0.001 based on a t-statistic determined from the degrees of freedom in the data. Standard deviation and difference maps were all calculated using IMOD.

### Docking of Crystal Structures into cryo-EM Density Maps

Docking of crystal structures into the 3D maps obtained from helical reconstruction was performed as described in Rank et al. [6]. The crystal structure of the αβ-tubulin dimer (PDB accession: 1JFF) [33] and the X-ray crystal structure of the ADP-state Kar3Vik1 heterodimer (SHD-Kar3Vik1E355C-K423C –EBI, PDB accession: 4ETP) [6] were docked manually into the density maps using UCSF Chimera. The ADP-state Kar3Vik1 heterodimer was docked as a single rigid-body unit into the maps manually by positioning Kar3 onto the MT based on the results of Hirose et al. [18]. Without any further adjustments, manipulation or flexible fitting, the Kar3Vik1 X-ray crystal structure fit excellently into the cryo-EM map of the wild-type GCN4-Kar3Vik1-microtubule complex in the nucleotide-free state (see Figure 4A and B in Rank et al., [6]). The fit could be improved by a small angular rotation of the coiled-coil stalk (see Fig. 5C) which may indicate a movement that occurs upon microtubule binding and/or loss of nucleotide from the Kar3 active site, or may simply be an artifact from the crystal packing.

To dock Kar3Vik1 into the AMPPNP state cryo-EM map, the X-ray crystal structure was divided into two rigid-body components that were manipulated separately: i) the Kar3 motor domain core (G385–T723), and ii) the complete Vik1 domain with the coiled-coiled stalk (Vik1 A320-Q638 and Kar3 A332-R384). These two rigid-body components were docked manually into the map by eye using UCSF Chimera. The best visual fit was obtained when the Kar3 motor domain remained docked at the microtubule as in the nucleotide-free state while Vik1 and the coiled-coil were rotated about Kar3 G385 to coincide with the positions of Vik1 and the coiled-coil in the wild-type GCN4-Kar3Vik1 AMPPNP state cryo-EM map (see Figure 4C and D in Rank et al., [6]).

To visualize the position of the Nanogold®-labeled cysteine residue utilized in our experiments here (Fig. 5C and D), the cryo-EM maps of wild-type GCN4-Kar3Vik1 from our docking experiments were replaced with the cryo-EM maps of Nanogold®-labeled GCN4-Kar3CFVik1C536C. Without additional movements or manipulations, the location of Vik1 C536 corresponds exceptionally well to the Nanogold density apparent in the cryo-EM maps in both the nucleotide-free and AMPPNP states (Fig. 5C and D). The absence of the stalk density from the AMPPNP state map in Fig. 5D is a result of the strong signal from the Nanogold® label that causes the stalk density to disappear at the map’s current threshold level.

## Public Data Deposition

CryoEM helical reconstructions maps have been deposited to the EMDB (http://emdatabank.org) with accession numbers EMD-5416 (GCN4-Kar3Vik1, nucleotide-free state) and EMD-5417 (GCN4-Kar3Vik1, AMPPNP state).

## Supporting Information

Figure S1 **Helical averages of Kar3Vik1-decorated MTs in three different nucleotide states.** The left panel shows 2D projections through the helical reconstructions of Kar3Vik1 bound to MTs in the nucleotide-free (A), AMPPNP (B), and ADP-AlF4- (C) states, with their corresponding Fourier transforms in the right panel.
(TIF)

Figure S2 **Plots of amplitudes and phases show averages comprise high-quality data.** The amplitudes (solid lines) and phases (dotted lines) of each of the layer lines of the final helical averages of Kar3Vik1 bound to MTs in the nucleotide-free (A) and AMPPNP (B) states. The amplitudes indicate the contribution of each layer line to the average. Relatively little scattering in the phase plots shows that the average is composed of good quality data.
(TIF)

Figure S3 **Phase plots reveal the resolution limit of the data.** Phase plots for three selected layer lines of all the individual datasets included in the final nucleotide-free (A) and AMPPNP (B) state helical reconstructions (each dot is one phase value). The clustering of datasets at certain phases reflects reliable data while areas with scattered phases are considered noise. The resolution limit of the 3D maps can thus be determined from these plots to be ∼ 2.5–2.2 nm.
(TIF)

Figure S4 **Fourier Shell Correlation curves provide an estimate of resolution of the helical reconstruction data.** The total data included in each final helical reconstruction was divided in half. Individual reconstructions were made from each of the half datasets and these two reconstructions were correlated against each other over a range of spatial frequencies. Using a correlation score of 0.5 as a criterion for estimating the resolution of the data, the helical averages presented in this paper have resolutions ranging from 2.2–2.5 nm. Fourier Shell Correlation curves were calculated and plotted using PEET.
(TIF)
