# Supplementary figures and images for: Kar3Vik1 Uses a Minus-End Directed Powerstroke for Movement along Microtubules
Source: PLoS One. 2013 Jan 14;8(1):e53792. doi: 10.1371/journal.pone.0053792 (PMC3544905; doi:10.1371/journal.pone.0053792)

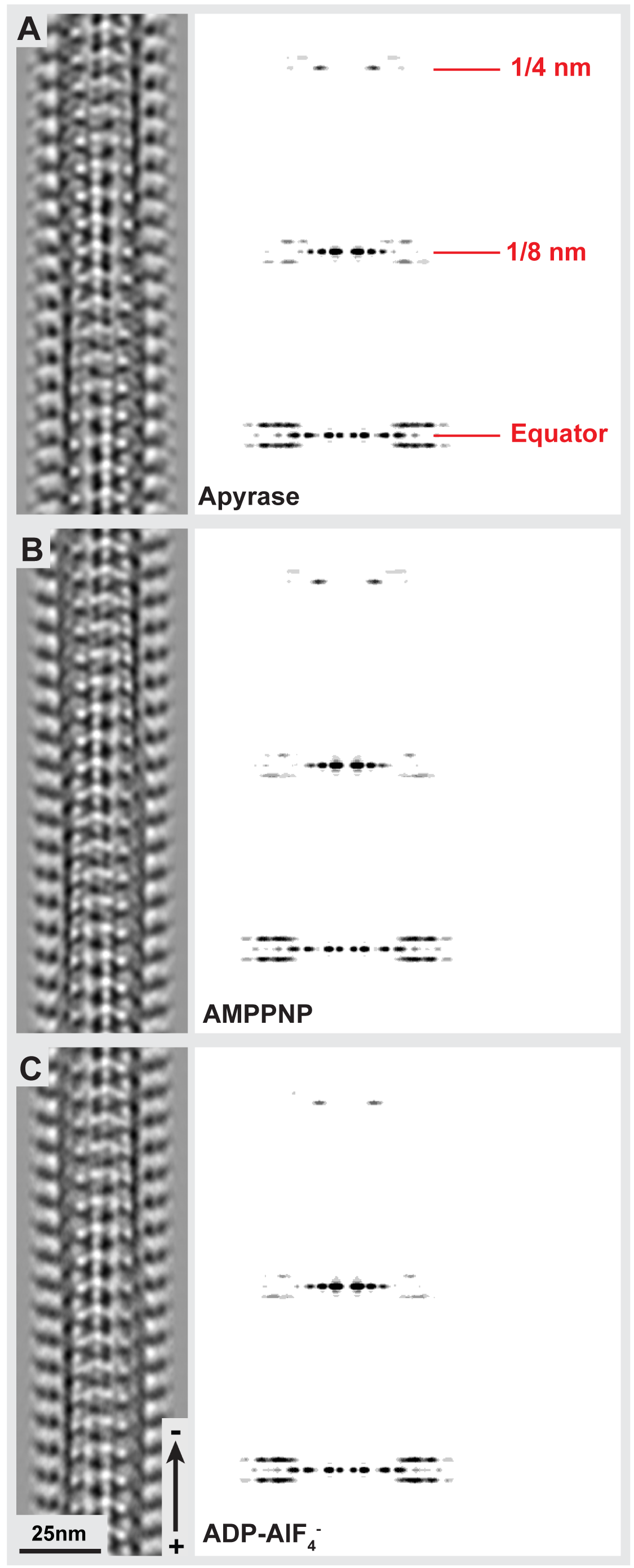

Supplement: Figure S1 — Helical averages of Kar3Vik1-decorated MTs in three different nucleotide states. The left panel shows 2D projections through the helical reconstructions of Kar3Vik1 bound to MTs in the nucleotide-free (A), AMPPNP (B), and ADP-AlF4- (C) states, with their corresponding Fourier transforms in the right panel. (TIF) [file pone.0053792.s001.tif]

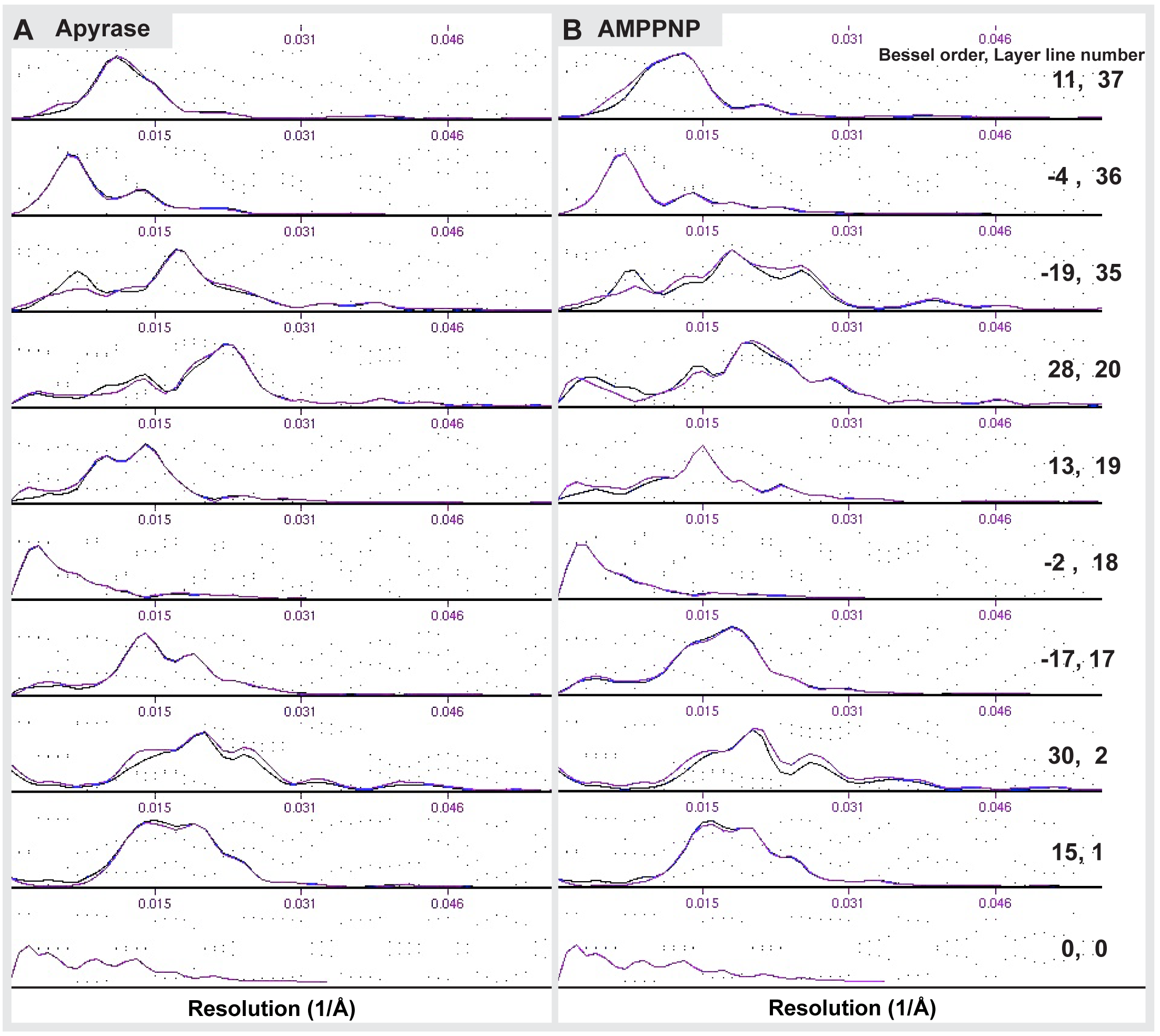

Supplement: Figure S2 — Plots of amplitudes and phases show averages comprise high-quality data. The amplitudes (solid lines) and phases (dotted lines) of each of the layer lines of the final helical averages of Kar3Vik1 bound to MTs in the nucleotide-free (A) and AMPPNP (B) states. The amplitudes indicate the contribution of each layer line to the average. Relatively little scattering in the phase plots shows that the average is composed of good quality data. (TIF) [file pone.0053792.s002.tif]

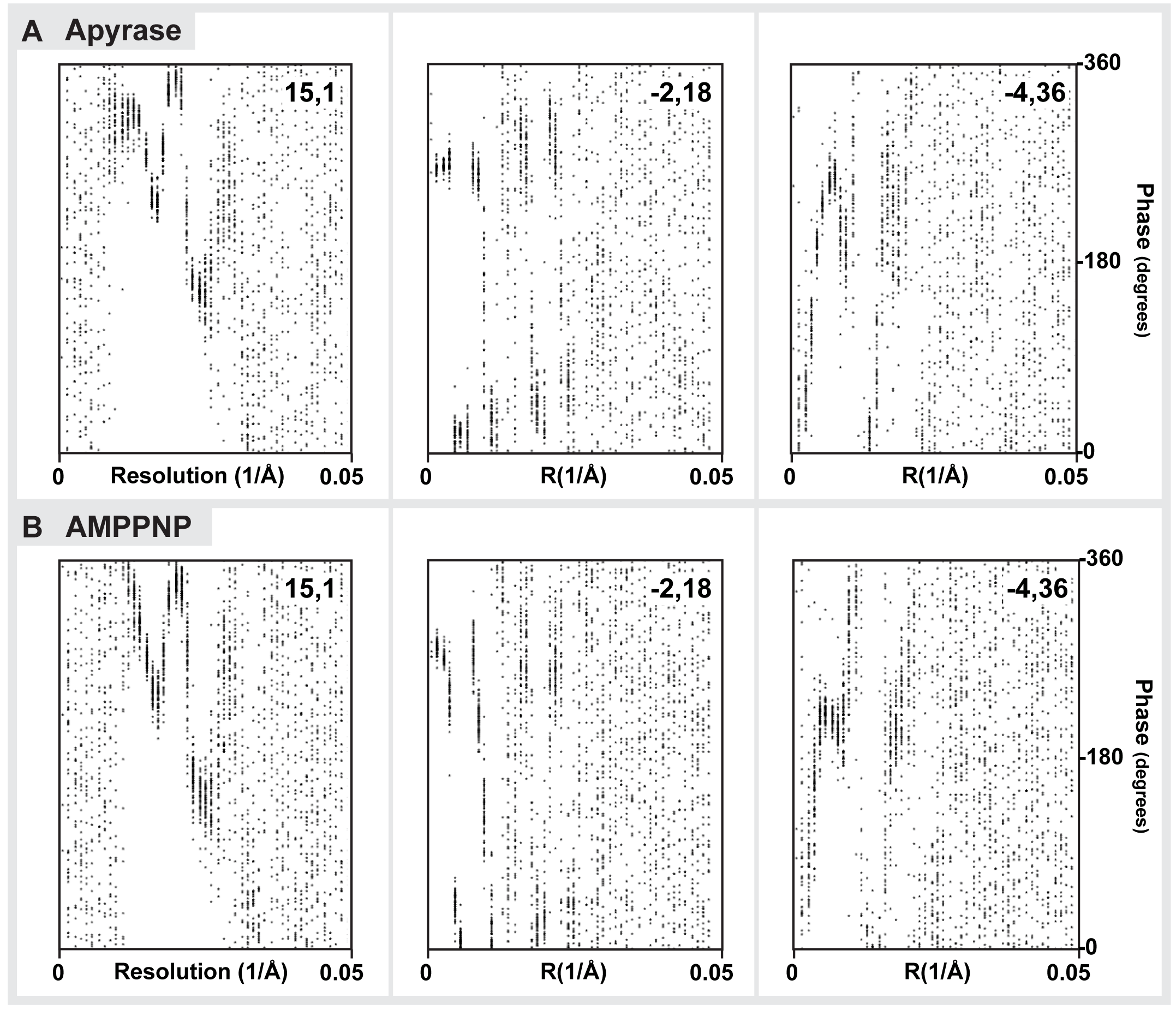

Supplement: Figure S3 — Phase plots reveal the resolution limit of the data. Phase plots for three selected layer lines of all the individual datasets included in the final nucleotide-free (A) and AMPPNP (B) state helical reconstructions (each dot is one phase value). The clustering of datasets at certain phases reflects reliable data while areas with scattered phases are considered noise. The resolution limit of the 3D maps can thus be determined from these plots to be ∼ 2.5–2.2 nm. (TIF) [file pone.0053792.s003.tif]

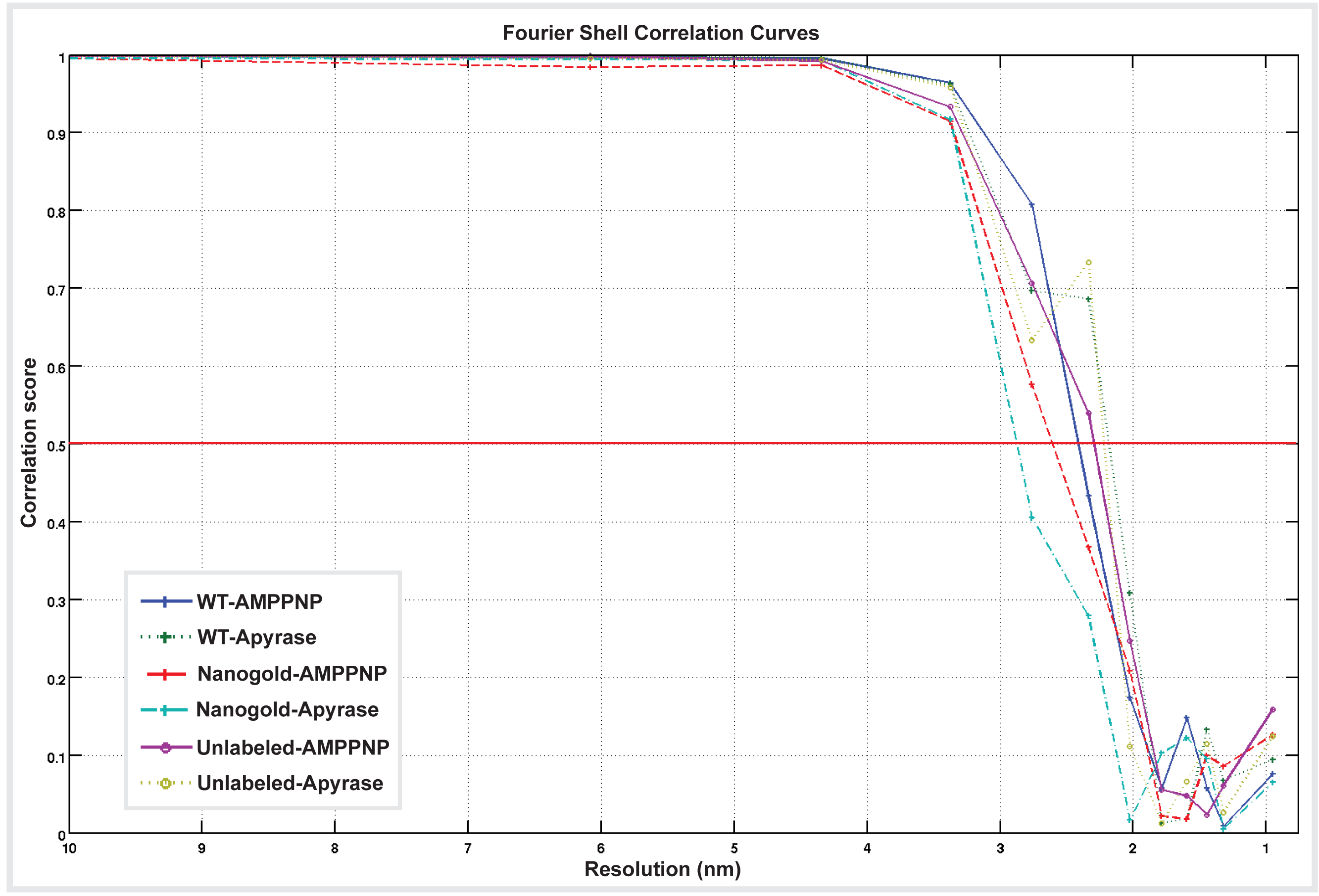

Supplement: Figure S4 — Fourier Shell Correlation curves provide an estimate of resolution of the helical reconstruction data. The total data included in each final helical reconstruction was divided in half. Individual reconstructions were made from each of the half datasets and these two reconstructions were correlated against each other over a range of spatial frequencies. Using a correlation score of 0.5 as a criterion for estimating the resolution of the data, the helical averages presented in this paper have resolutions ranging from 2.2–2.5 nm. Fourier Shell Correlation curves were calculated and plotted using PEET. (TIF) [file pone.0053792.s004.tif]
